# Working Memory-Related Effective Connectivity in Huntington’s Disease Patients

**DOI:** 10.3389/fneur.2018.00370

**Published:** 2018-06-04

**Authors:** Jacob Lahr, Lora Minkova, Sarah J. Tabrizi, Julie C. Stout, Stefan Klöppel, Elisa Scheller, the TrackOn-HD Investigators, A. Coleman

**Affiliations:** ^1^Department of Psychiatry and Psychotherapy, Faculty of Medicine, University Medical Center Freiburg, Freiburg, Germany; ^2^Freiburg Brain Imaging Center, Faculty of Medicine, University Medical Center Freiburg, Freiburg, Germany; ^3^Department of Neurodegenerative Disease, Institute of Neurology, University College London, London, United Kingdom; ^4^School of Psychological Sciences, Institute of Clinical and Cognitive Neuroscience, Monash University, Melbourne, VIC, Australia; ^5^Center for Geriatric Medicine and Gerontology, Faculty of Medicine, University Medical Center Freiburg, Freiburg, Germany; ^6^University Hospital of Old Age Psychiatry and Psychotherapy, University of Bern, Bern, Switzerland; ^7^Department of Psychology, Laboratory for Biological and Personality Psychology, University of Freiburg, Freiburg, Germany

**Keywords:** functional magnetic resonance, Huntington’s disease, dynamic causal modelling, effective connectivity, cluster analysis, working memory, *n*-back

## Abstract

Huntington’s disease (HD) is a genetically caused neurodegenerative disorder characterized by heterogeneous motor, psychiatric, and cognitive symptoms. Although motor symptoms may be the most prominent presentation, cognitive symptoms such as memory deficits and executive dysfunction typically co-occur. We used functional magnetic resonance imaging (fMRI) and task fMRI-based dynamic causal modeling (DCM) to evaluate HD-related changes in the neural network underlying working memory (WM). Sixty-four pre-symptomatic HD mutation carriers (preHD), 20 patients with early manifest HD symptoms (earlyHD), and 83 healthy control subjects performed an *n*-back fMRI task with two levels of WM load. Effective connectivity was assessed in five predefined regions of interest, comprising bilateral inferior parietal cortex, left anterior cingulate cortex, and bilateral dorsolateral prefrontal cortex. HD mutation carriers performed less accurately and more slowly at high WM load compared with the control group. While between-group comparisons of brain activation did not reveal differential recruitment of the cortical WM network in mutation carriers, comparisons of brain connectivity as identified with DCM revealed a number of group differences across the whole WM network. Most strikingly, we observed decreasing connectivity from several regions toward right dorsolateral prefrontal cortex (rDLPFC) in preHD and even more so in earlyHD. The deterioration in rDLPFC connectivity complements results from previous studies and might mirror beginning cortical neural decline at premanifest and early manifest stages of HD. We were able to characterize effective connectivity in a WM network of HD mutation carriers yielding further insight into patterns of cognitive decline and accompanying neural deterioration.

## Introduction

Huntington’s disease (HD) is a genetically caused progressive neurodegenerative disorder characterized by a combination of motor, cognitive, and psychiatric symptoms. It is caused by a cytosine–adenine–guanine (CAG) trinucleotide repeat expansion in the huntingtin gene that can be diagnosed years before the onset of first symptoms. The number of CAG repeats is the strongest predictor for age and probability of onset. In patients with 40 or more CAG repeats, the disease is fully penetrant, and symptoms will occur.

Working memory (WM) is one of the first cognitive domains to be impaired in HD patients and is already affected in preclinical HD mutation carriers (1, 2). Verbal WM tasks robustly activate a fronto-parietal network (3), which is prone to alterations in aging and in neurodegenerative disease (4, 5). Therefore, the fronto-parietal network has been investigated in task functional magnetic resonance imaging (fMRI) studies in HD patients over the last years [for reviews, see Ref. (6, 7)], identifying complex patterns of HD disease-specific hyper- and hypoactivations in key brain areas involved in WM-related tasks, such as the dorsolateral prefrontal cortex (DLPFC), parietal cortex, and striatum (8–11). With regard to correlation-based functional connectivity, Wolf et al. (10) found decreased WM-related functional connectivity in left fronto-striatal and fronto-parietal networks in preHD subjects. In the IMAGE-HD study, there was no such clear left lateralization in a larger sample of mutation carriers. In a longitudinal design, reduced connectivity from right DLPFC to parietal cortex [over 18 months (12)] and reduced connectivity between left DLPFC and caudate [over 30 months (13)] were observed.

To disambiguate these variable findings with regard to activity and connectivity changes, a more sophisticated analysis method is warranted. Dynamic causal modeling (DCM) provides an ideal framework to investigate directed causal interactions within a predefined network of brain areas, such as the fronto-parietal WM network (14). It has successfully been used to demonstrate effective connectivity alterations in clinical groups, such as schizophrenia patients showing impairments in prefrontal–parietal connectivity during a WM task (15, 16). Similarly, we have previously used an fMRI motor task to show that DCM connectivity measures were predictive of disease progression in individuals with HD before clinical onset (17). Furthermore, in a previous study based on the large-scale, multicenter TrackOn-HD study (18), we focused on compensation mechanisms in preHD mutation carriers, with DCM analyses based on resting-state fMRI. Our findings were consistent with compensation characterized by increased functional coupling between the right dorsolateral prefrontal cortex (rDLPFC) and a left hemisphere network as assessed by rsfMRI, which predicted better cognitive performance despite the presence of brain atrophy (18). Interestingly, no indication of compensation was found for the motor network previously defined in an independent cohort (17, 19). Our further analyses with the TrackOn-HD data aimed at investigating effective connectivity in the motor network by also including manifest HD (20), where we were able to demonstrate stratification of HD patients using a hierarchical cluster analysis based on DCM.

To extend on our previous findings, we here use a similar DCM approach based on task fMRI to evaluate effective connectivity in the WM network in a large sample of HD patients, HD mutations carriers, and healthy controls (HCs). In addition, we investigated the association of behavioral performance, structural markers of disease progression, and CAG-repeat length with DCM parameters during the WM task. Finally, we performed a cluster analysis to further investigate if measures of effective connectivity within the WM network allow identifying subgroups of gene mutations carriers that would be related to the clinically often strikingly heterogeneous pattern of manifestation, considering the monogenetic cause.

## Materials and Methods

The study design was adopted from our previous study on DCM characteristics in a motor task in the TrackOn-HD study population (20). For reasons of contingency and comparability, we aimed to use the same methods and parameters where feasible.

### Participants

A total of 241 participants were recruited within the large-scale, multimodal TrackOn-HD study at four different sites (Paris, London, Vancouver, and Leiden). Twenty-two subjects were excluded as they did not participate in the verbal WM task. Other exclusion criteria included technical issues (*n* = 1), corrupt or missing fMRI data (*n* = 6), poor task performance (*n* = 9) and missing activations (*n* = 6), as well as failed DCM quality check (*n* = 30; see DCM Analysis), finally yielding 167 datasets to be analyzed. There was no significant effect between-group affiliation (HCs: 29 exclusions, mutation carriers: 29 exclusions or early manifest HD: 16 exclusions) and number of excluded participants [χ^2^(2, *N* = 241) = 4.42, *p* = 0.11]. In a previous study on behavioral data from the Track-HD study (21), considerable practice effects have been shown between Visit 1 and Visit 2. Therefore, aiming for low variability, we analyzed data from Visit 2 (out of three visits in the TrackOn-HD study), where participants and personnel were already acquainted with the tasks.

Thus, the final dataset consisted of 167 participants scanned between April and November 2013, comprising the following three groups: 64 individuals without HD but carrying the mutant huntingtin gene (preHD: 28 females, mean age ± SD: 42.12 ± 8.75 years), 20 early manifest HD patients (earlyHD: 10 females, mean age ± SD: 43.89 ± 5.96 years), and 83 HC subjects (HC: 48 females, mean age ± SD: 49.11 ± 10.33 years). preHD required a disease burden of pathology score greater than 250 and a total motor score of 5 or less in the motor assessment of the Unified Huntington’s Disease Rating Scale 99 [UHDRS (22)], indicating no substantial motor signs. earlyHD were required to have motor symptoms consistent with HD, and a diagnostic confidence score of 4, according to the UHDRS, as well as to be within the Shoulson and Fahn stage I or II (23) assessed according to UHDRS total functional capacity (TFC ≥ 7) (24). Age, level of education [as measured by the International Standard Classification of Education (25)], gender, and site were considered as covariates in all analyses. Caudate volume (adjusted for total intracranial volume), disease burden score [(DBS) (26)], and cumulative probability of clinical onset [CPO (27)] were used as markers of HD disease progression. Demographic and clinical data were compared across groups using ANCOVA (see Table 1).

**Table 1:** Demographic and clinical information.

	HC (*n* = 83)	preHD (*n* = 64)	earlyHD (*n* = 20)
Age (years)^a^	49.11 ± 10.33	42.12 ± 8.75	43.89 ± 5.96
Gender (f/m)	48/35	28/36	10/10
Handedness (right/left/both)	76/5/2	59/1/4	18/2/0
Education (ISCED)	4.00 ± 1.02	4.05 ± 1.10	4.00 ± 0.79
CAG repeats^a^	–	43.08 ± 2.41	43.90 ± 1.77
CPO^a^	–	0.21 ± 0.14	0.42 ± 0.18
Disease burden score^a^	–	302 ± 51	362 ± 56
Caudate^a^ (TIV adjusted)	0.52 ± 0.04	0.44 ± 0.06	0.36 ± 0.07

*Values are given as mean ± SD, where applicable. HC, healthy control; preHD, pre-symptomatic HD mutation carrier; earlyHD, early manifest HD patient; f, female; m, male; CPO, cumulative probability of clinical onset, DBS = age × (CAG-length − 35.5) (26); TIV, total intracranial volume; CAG, cytosine–adenine–guanine; ISCED, International Standard Classification of Education*.

*^a^Significant differences between groups. Please refer to Section “Results” for more information*.

The study was approved by the Ethics Committees of the Institute of Neurology, UCL (London), the University of British Columbia (Vancouver), Pierre and Marie Curie University (Paris), and the University of Leiden (Leiden). All participants gave a written informed consent according to the Declaration of Helsinki (28).

### Verbal Working Memory fMRI Paradigm

**Figure 1:**
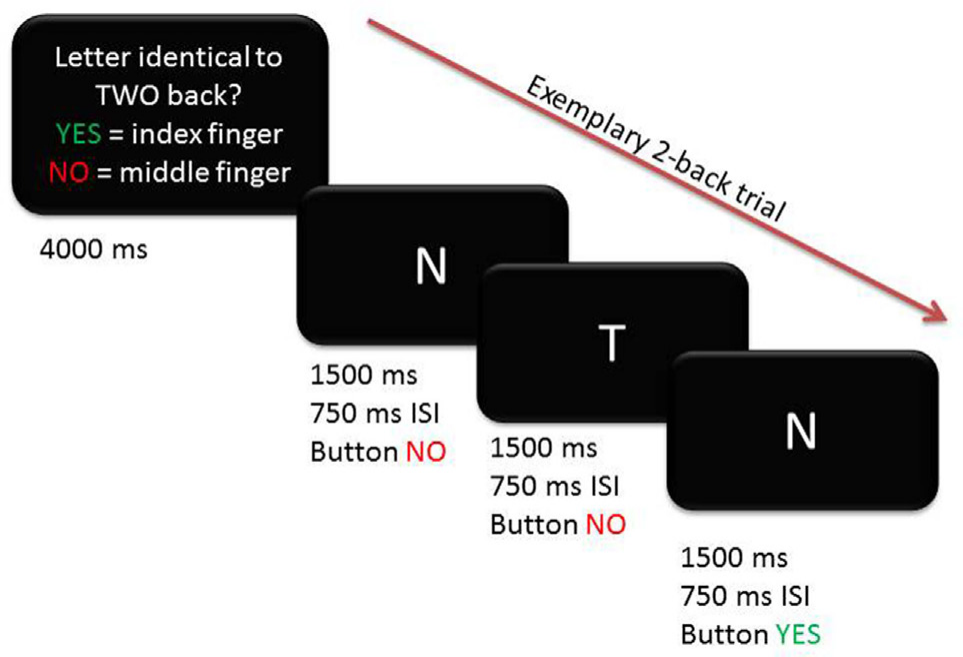
Exemplary trial for 2-back condition depicting timing and correct button presses.

Participants underwent fMRI scanning while performing a blocked verbal *n*-back task with two levels of WM load (1-back and 2-back, Figure 1). A third condition where participants had to indicate whether the letter A was presented (0-back) was used as baseline to contrast against 1-back and 2-back conditions. Participants responded with their right index (indicating “yes” or “match”) and middle finger (indicating “no” or “no match”) using a two-button response box. Before scanning, participants were given instructions outside of the scanner and practiced each condition first outside, then inside the scanner. A rate of at least 70% correct responses in the 1-back training condition was required before starting the task to ensure that participants had understood the task. Instructions and letters were presented in light gray against a black background with font size scaled according to the imaging site-specific mirror-projector setup. The three conditions were presented in a blocked design in a pseudo-randomized order. At the beginning of each block, condition-specific instructions in the respective spoken language were presented on the screen for 4 s. There were 6 blocks per condition, each lasting 30 s during which 10 letters were displayed. Stimuli were presented for 1,500 ms with a 750 ms interstimulus interval. Performance in the 1-back and 2-back conditions was assessed using both the d-prime coefficient (probability of correct response minus probability of false positive responses) and reaction time and analyzed across groups and conditions using an ANCOVA, adjusting for age, gender, site, and education.

### MRI Data Acquisition and fMRI Analysis

A standard general linear model (GLM) analysis of the fMRI paradigm with data from Visit 1 has been reported previously (18), for more detailed information on the GLM analysis, please refer to the latter study. Participants were scanned on 3 T Siemens MAGNETOM TimTrio MR scanners at Paris and London and on 3 T Philips Achieva MR scanners at Vancouver and Leiden. High-resolution three-dimensional T1-weighted structural scans were acquired for all participants to exclude structural abnormalities not related to HD. For the fMRI WM task, 225 whole-brain volumes were acquired using a T2*-weighted single-shot gradient echo planar imaging (GE-EPI) sequence with the following parameters: TR = 3,000 ms, TE = 30 ms, FOV = 212 mm, flip angle = 80°, 48 slices in ascending order (slice thickness: 2.8 mm, gap: 1.5 mm, in plane resolution 3.3 mm × 3.3 mm). Data preprocessing was performed in SPM8 (29) as in the initial report (18).

Statistical analysis at the first (within-subject) level was carried out using the GLM in SPM8. Task-related changes of BOLD signals were estimated at each voxel by modeling each block separately for each of the conditions (0-back, 1-back, and 2-back). Subject-specific contrasts of interest were created from the beta estimates coding the effect of WM load (0-back, 1-back, and 2-back). Main effects of experimental task were characterized using one-sample *t*-tests, including age, gender, education, and site as confounding covariates as implemented in SPM. All participants were included in the one-sample *t*-tests as one group to ensure that regions of interests (ROIs) for the subsequent DCM analysis were commonly activated across all groups. Task-specific activations were identified at *p* < 0.05 FWE corrected. In addition, between-group comparisons were implemented using a 3 × 3 ANCOVA design, including group (HC, preHD, and HD) as a between-group factor, as well as WM load (2-back, 1-back, and 0-back) as a within-group factor, while correcting for age, gender, site, and education.

### DCM Analysis

Effective connectivity analysis was conducted using DCM (30), a hypothesis-driven Bayesian approach that describes the biophysical nature of directed interactions among distinct brain regions by incorporating two forward models: one at the neural and one at the hemodynamic level. By combining *a priori* knowledge of a biologically plausible neural model (input) with the measured BOLD response (output), it is possible to infer on underlying hidden states such as regional causal interactions.

Identical to our earlier analyses (18, 20), we used deterministic, bilinear, one-state DCM to assess the effective connectivity among five regions of the WM network [Owen et al. (3) and Table 2]. These regions comprised the left and right inferior parietal cortex (IPC), left anterior cingulate cortex (ACC), as well as left and right dorsolateral prefrontal cortices (DLPFCs). The activation pattern evoked by the contrast 2-back vs. 0-back provided evidence for the choice of intrinsic connections between the five ROI. Furthermore, the differential effect of WM load (as expressed by the 2-back vs. 1-back contrast) motivated the choice of task-modulated connections.

**Table 2:** Behavioral results from the working memory task (mean and SD).

	HC	preHD	earlyHD
d-prime 1-back	4.03 ± 0.61	3.80 ± 0.83	3.44 ± 1.02
d-prime 2-back	2.68 ± 0.96	2.66 ± 0.94	2.06 ± 1.00
rt (ms) 1-back	803 ± 163	863 ± 204	998 ± 246
rt (ms) 2-back	958 ± 218	1,000 ± 227	1,127 ± 229

For each participant, time series from each of the five ROIs were extracted using the fixed coordinates from the second-level activations identified in the one-sample *t*-test and adjusted for the effect of interest (*F*-contrast). No statistical threshold was used within each ROI, which allowed for the time series extraction of the same set of voxels in all participants. The motivation for this approach is based on previous literature (31) and is advantageous for this study because it ensured that there was no overlap of subject-specific spheres in neighboring brain regions. Furthermore, participants having ROIs with weak activations do not have to be excluded but at the expense of potentially including condition-independent noise (31). This is an issue particularly in small sample sizes but potentially less so in our relatively large study.

**Figure 2:**
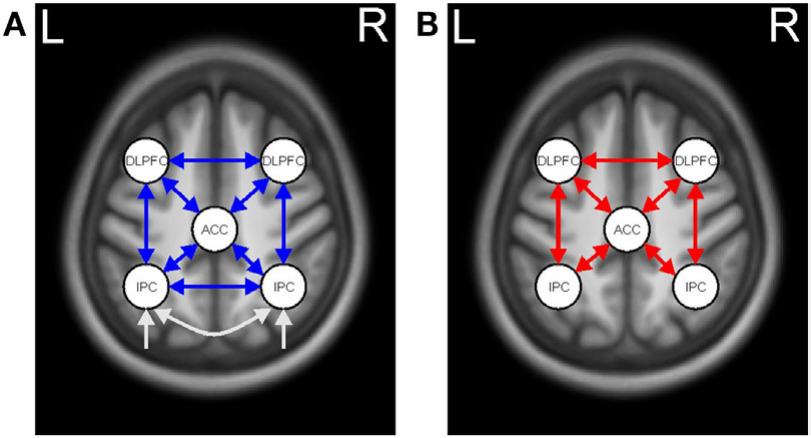
Dynamic causal model for working memory (WM). **(A)** Task-independent, intrinsic connections (blue arrows) and driving input (white arrows). **(B)** WM-modulated connections (red arrows).

The extracted time series of all five ROIs were included in one fully connected DCM model, and intrinsic connections were modeled among these regions (see Figure 2A). The fully connected DCMs were then reduced using the *post hoc* optimization procedure for approximating model evidence, proposed by Friston and Penny (32). This approach optimizes only the fully connected model, while the evidence for any sub-model is obtained using generalization of the Savage–Dickey density ratio (33). In addition, *post hoc* diagnostics of each participant’s DCM were conducted using in-house MATLAB routines (adapted from https://sites.google.com/site/jeandaunizeauswebsite/code/explore-dcm) to ensure that model inversion had converged, requiring at least 10% of variance explained.

Dynamic causal modeling model specification, estimation, and *post hoc* optimization were carried out with DCM12, as implemented in SPM12b. Statistical inference on model parameters was conducted in SPSS, Version 22.0 (IBM Corporation, NY, USA). Random-effects inference at the connection level was assessed using ANCOVA analysis after covariate adjustment. Between-group differences were considered significant at a threshold of *p* < 0.001 after accounting for the number of connections (i.e., 21 intrinsic and 14 modulatory). Pairwise comparisons were used for *post hoc* analyses of significant between-group differences, applying Bonferroni correction for the three groups.

### Correlation and Cluster Analysis

We used Pearson’s partial correlation analysis among HD mutation carriers, including age, gender, site, and education as covariates of no interest, to examine how DCM parameters were correlated with behavioral performance, CAG-triplet expansion, and caudate volume as a marker of disease progression. Bonferroni correction was used to account for the number of correlation tests.

To identify subgroups differing in connectivity pattern, DCM intrinsic and modulatory parameters across all HD gene mutation carriers (preHD and earlyHD) were entered into a hierarchical agglomerative cluster analysis, as implemented in SPSS. Ward’s clustering linkage method (34) was performed on all parameters with squared Euclidean distance as a measure of proximity. We used the agglomeration schedule (i.e., the change in agglomeration coefficients as the number of clusters increase) to determine the optimum number of clusters. In case of a meaningful clustering, each HD mutation carrier is assigned to one of the identified subgroups by repeating the cluster analysis using the optimal number of clusters. Finally, Pearson’s partial correlation analysis, including age, gender, site, and education as covariates of no interest, was used to examine how subgroup membership was correlated with behavioral performance and caudate volume as a marker of disease progression.

## Results

### Clinical Measures

ANCOVA of caudate volume showed a significant difference between groups [*F*(2, 159) = 85.29, *p* < 0.001]. Pairwise *post hoc* comparisons revealed significant differences between all groups (*p* < 0.001) with the largest caudate volume in HC, followed by preHD and earlyHD. There was no difference in the level of education between groups [*F*(2, 161) = 0.03, *p* = 0.97]. After exclusions, there was a significant effect of age between groups [*F*(2, 161) = 9.78, *p* < 0.001]. *Post hoc* testing revealed that this effect was driven by a significant difference in age between HC and preHD (*p* < 0.001) as well as between HC an earlyHD (*p* = 0.049). ANCOVA indicated that CPO [*F*(1, 78) = 35.32, *p* < 0.001], DBS [*F*(1, 78) = 27.17, *p* < 0.001] as well as number of CAG repeats [*F*(1, 78) = 12.67, *p* = 0.001] were higher in the earlyHD group than in the preHD group.

### Behavioral Data

Repeated measures ANCOVA with WM load as a within-subject factor and group (HC, preHD, and earlyHD) as between-subject factor, as well as age, gender, site, and education as covariates, showed a significant main effect of WM load on task performance [d-prime, *F*(1, 160) = 19.85, *p* < 0.001] and on reaction time [*F*(1, 160) = 18.05, *p* < 0.001], as well as a significant effect of group [d-prime, *F*(2, 160) = 7.43, *p* = 0.001; reaction time, *F*(2, 160) = 9.45, *p* < 0.001]. Pairwise *post hoc* comparisons revealed significant differences in d-prime between HC and earlyHD (*p* = 0.001), as well as between preHD and earlyHD (*p* = 0.043), and in reaction time between HC and preHD (*p* = 0.016) and between HC and early HD (*p* < 0.001). Interactions between WM load and group were not significant. Descriptive information on WM performance is provided in Table 2.

### fMRI Results

Performing the WM task was associated with substantial BOLD signal increases in the well-established fronto-parietal WM network (3), basal ganglia, and thalamus for controls, preHD participants and earlyHD patients in the contrast 2-back vs. 0-back. Whole-brain analyses of group differences (taking into account, age, gender, site, and education as covariates) did not reveal increased activation in both WM conditions (1-back and 2-back) in preHD and earlyHD compared with HC at a statistical threshold of *p* < 0.05 FWE corrected. The same applied to the reverse contrasts assessing increased activation in HC compared with preHD and earlyHD.

### DCM Results

#### *Post Hoc* Optimization

*Post hoc* analysis resulted in the same winning model across the three groups with the highest probability of (almost) 1. In the winning model, neither intrinsic nor modulatory connections were removed (Figure 2). A separate analysis for mutation carriers and controls revealed the same winning model after the *post hoc* optimization procedure. The posterior probabilities were further examined using a one-sample *t*-test against 0 (see Table 3 for descriptive statistics).

**Table 3:** Descriptive statistics of dynamic causal modeling connection strengths.

	HC (*n* = 83)		preHD (*n* = 64)		earlyHD (*n* = 20)	
	Mean	SD	Mean	SD	Mean	SD
**Intrinsic connections (A-matrix)**						
lIPC to lIPC	0.032	0.251	−0.095*	0.165	−0.076	0.331
lIPC to rIPC	0.029	0.338	0.031	0.297	0.074	0.223
lIPC to lACC	−0.149*	0.318	0.036	0.332	−0.080	0.174
lIPC to lDLPFC	0.062	0.277	−0.136*	0.266	−0.018	0.282
rIPC to lIPC	−0.053	0.173	0.069	0.215	−0.069	0.255
rIPC to rIPC	0.012	0.240	−0.081*	0.156	0.159	0.262
rIPC to lACC	−0.178*	0.273	0.031	0.249	0.103	0.276
rIPC to rDLPFC	0.141*	0.295	−0.133*	0.213	−0.216*	0.212
lACC to lIPC	0.206*	0.224	0.270*	0.206	0.061	0.259
lACC to rIPC	−0.079*	0.215	0.210*	0.199	0.001	0.318
lACC to lACC	−0.072*	0.173	−0.149*	0.127	0.082	0.295
lACC to lDLPFC	−0.107*	0.192	−0.035	0.183	0.085	0.193
lACC to rDLPFC	0.249*	0.275	−0.158*	0.222	−0.144	0.208
lDLPFC to lIPC	0.099*	0.160	0.297*	0.195	0.043	0.266
lDLPFC to lACC	−0.064*	0.137	0.081*	0.173	0.164*	0.188
lDLPFC to lDLPFC	−0.058*	0.152	−0.090*	0.103	−0.001	0.119
lDLPFC to rDLPFC	0.178*	0.156	−0.010	0.162	0.064	0.119
rDLPFC to rIPC	0.080*	0.152	0.220*	0.202	0.010	0.214
rDLPFC to lACC	0.066*	0.120	0.086*	0.154	0.312	0.793
rDLPFC to lDLPFC	−0.061*	0.160	0.068	0.164	−0.095	0.985
rDLPFC to rDLPFC	0.365*	0.720	−0.118*	0.112	0.097	0.705

**Modulatory connections (B-matrix)**						
lIPC to lACC	0.099	0.825	0.331	0.947	0.204	0.824
lIPC to lDLPFC	0.436*	0.643	−0.222	1,066	0.445	0.818
rIPC to lACC	0.173	0.720	0.138	0.690	0.462	0.763
rIPC to rDLPFC	0.377*	0.858	0.127	0.704	−0.364	0.820
lACC to lIPC	0.412*	0.631	0.412*	0.842	0.073	0.864
lACC to rIPC	0.234	0.781	0.223	0.831	0.381	0.912
lACC to lDLPFC	−0.016	0.794	−0.098	0.757	0.112	0.806
lACC to rDLPFC	0.286	0.798	0.041	0.730	−0.408	0.831
lDLPFC to lIPC	0.447*	0.672	0.110	0.808	0.008	0.720
lDLPFC to lACC	0.010	0.732	0.217	0.789	0.185	0.766
lDLPFC to rDLPFC	0.040	0.831	−0.131	0.757	−0.002	0.572
rDLPFC to rIPC	0.205	0.596	−0.078	0.875	−0.269	0.458
rDLPFC to lACC	0.035	0.584	0.099	0.549	0.069	0.352
rDLPFC to lDLPFC	−0.067	0.477	0.012	0.602	−0.077	0.408

Connections that significantly differed from 0 (based on one-sample *t*-tests with a significance threshold *p* < 0.0014 after Bonferroni correction for 35 comparisons) are marked with an asterisk “*.”

*HC, healthy control; preHD, pre-symptomatic HD; earlyHD, early manifest HD; IPC, inferior parietal cortex; ACC, anterior cingulate cortex; DLPFC, dorsolateral prefrontal cortex*.

#### Differences in Task-Independent Coupling

**Figure 3:**
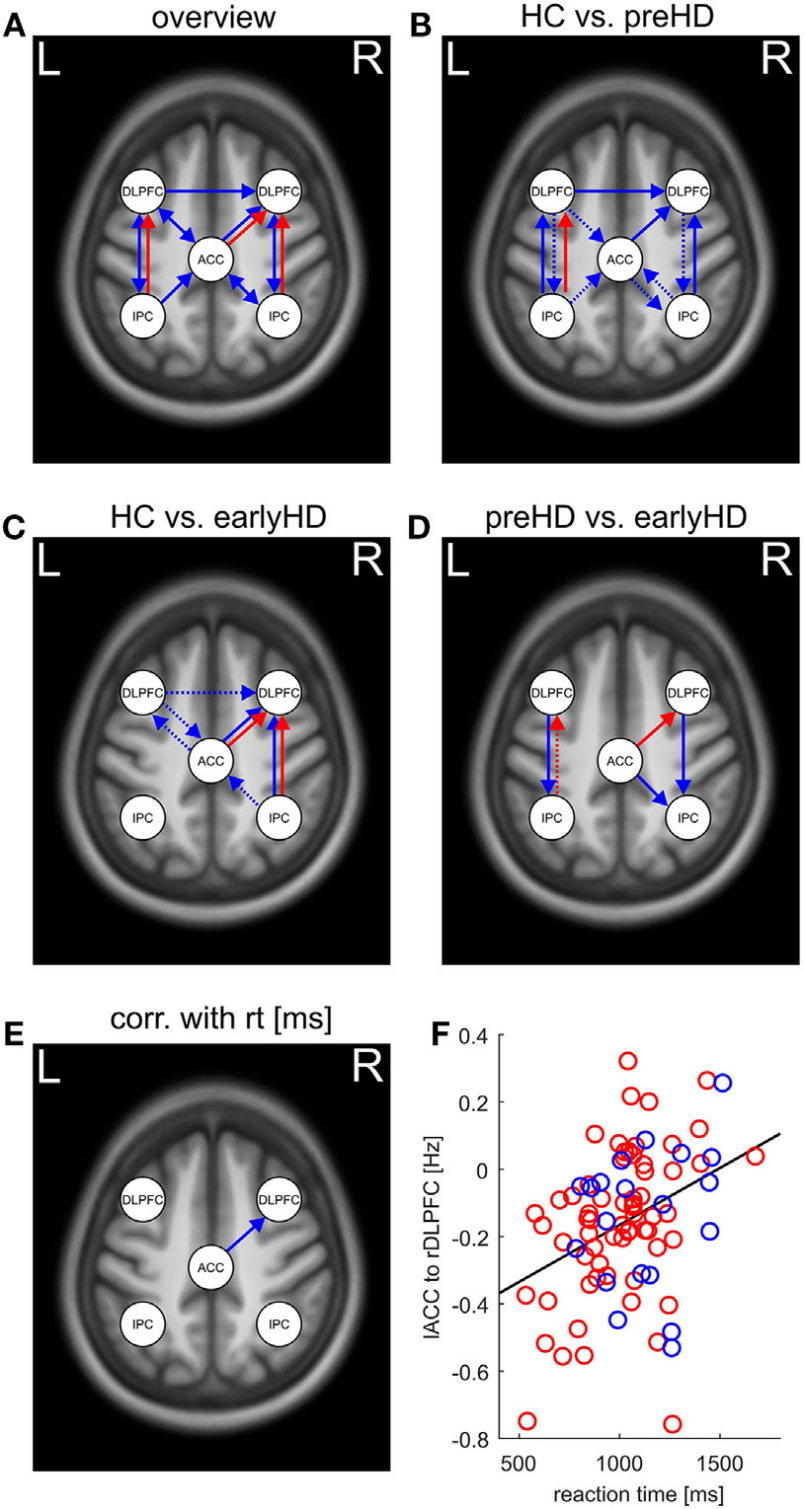
**(A)** Overview of between-group differences in intrinsic (blue arrows) and task-related (red arrows) effective connectivity (*p* < 0.0014 after Bonferroni correction for 35 comparisons) as revealed by ANCOVA. **(B–D)** Pairwise comparisons and direction of differences in effective connectivity as revealed by *post hoc* tests (dotted arrows indicate decreased connectivity; solid arrows indicate correspondingly increased connectivity. “Decreased” and “increased” connectivity refers to the group that is named first, respectively). **(B)** Pairwise comparisons between healthy control (HC) and preHD, **(C)** pairwise differences between HC and earlyHD, and **(D)** pairwise comparisons between preHD and earlyHD. **(E)** Positive association between response time in the 2-back task and intrinsic connectivity as revealed by partial correlation analysis. **(F)** Scatter plot of the reaction time in the 2-back task and intrinsic connectivity between left anterior cingulate cortex (lACC) and right dorsolateral prefrontal cortex (rDLPFC), preHD participants are represented in red, and earlyHD patients in blue, respectively.

Differences in effective connectivity among HC, preHD, and early-HD patients were found for 14 connections using ANCOVA (Table S1 in Supplementary Material) and are pictured in Figure 3A. Between-group differences in intrinsic connections were identified across the whole network. Specifically, preHD showed differential connectivity compared with HC across almost the entire network (Figure 3B). They had weaker intrinsic connectivity predominantly toward right DLPFC as well as from left IPC to left DLPFC. Increased intrinsic connectivity in preHD compared with HC was found mostly in the opposite direction from both DLPFC to other areas as well as from bilateral IPC to ACC. Differences in both intrinsic as well as task-related connectivity between HC and earlyHD showed a lateralization in the right hemisphere (Figure 3C). Interestingly, both types of connections from ACC and right IPC toward rDLPFC were decreased in earlyHD vs. HC, potentially pointing to intrinsic and task-related impairment of right DLPFC connectivity. The remaining significant intrinsic connections in this comparison were increased in earlyHD, mostly involving the left DLPFC (Figure 3C). Intrinsic connections differing between preHD and earlyHD (Figure 3D) included all anterior–posterior connections toward bilateral IPC. Here, earlyHD patients showed a decrease in connectivity, pointing to reduced coupling toward the posterior part of the WM network in manifest stages of HD.

#### Differences in Modulatory Coupling

Between-group differences in task-related connections were only identified in those toward bilateral DLPFC (Figure 3B). Here, preHD showed decreased coupling from left IPC to left DLPFC with increased WM load, potentially signifying lesser recruitment of left DLPFC with incremental task difficulty. A comparable effect can be observed in earlyHD compared with HC, although it emerged in the right hemisphere and concerned connections from both ACC and right IPC toward right DLPFC (Figure 3C). Together with the aforementioned decrease in the corresponding intrinsic connections, right DLPFC seemed to be only weakly connected to the rest of the WM network in earlyHD. Finally, the comparison of task-related connections between preHD and earlyHD revealed a decrease in coupling from left IPC to left DLPFC in preHD and an increase in coupling from ACC to right DLPFC in preHD compared with earlyHD (Figure 3D). Thus, earlyHD recruited right DLPFC to a lesser extent when task difficulty increased, paralleling the comparison with HC in Figure 3C: Even when compared with premanifest mutation carriers, patients in the manifest stage of the disease showed significantly weaker task-related coupling toward right DLPFC.

### Correlation and Cluster Analysis Results

To complement the between-group comparisons, markers of disease progression and behavioral parameters were correlated with measures of effective connectivity across mutation carriers. After Bonferroni correction for 35 comparisons, only the association between the reaction times in the 2-back task and the intrinsic connectivity between the left ACC and the right DLPFC remained significant (*r* = 0.36, *p* < 0.001, Figures 3E,F). Thus, stronger intrinsic connectivity from ACC to right DLPFC was associated with slower response in mutation carriers.

**Figure 4:**
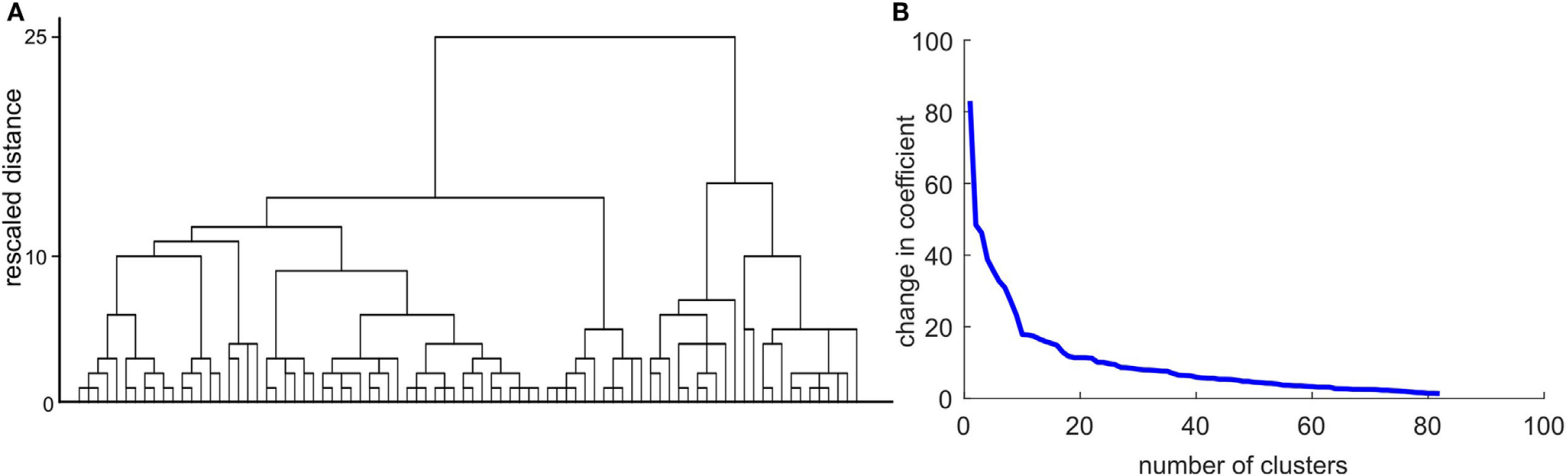
Results of the cluster analysis with intrinsic and task-related dynamic causal modeling parameters. **(A)** Dendrogram using Ward linkage. **(B)** The scree plot shows a potential inflection at *n* = 10.

After entering all intrinsic and task-related connectivity parameters of HD mutation carriers (preHD and earlyHD) into a hierarchical cluster analysis, we tried to identify the optimal number of clusters to define potential subgroups among mutation carriers. Unfortunately, both the resulting scree plot ands the dendrogram (Figure 4) did not allow obtaining a meaningful number of clusters. Hence, we refrained from conducting further analysis steps as described in Section “Materials and Methods,” since the results would not have yielded meaningful interpretation.

## Discussion

In this study, we explored effective connectivity in HD mutation carriers using DCM. To this end, we examined a uniquely large sample from the TrackOn-HD study, conducted a comprehensive analysis of connectivity, and herewith present the first task-based, WM-related DCM study in HD.

### Behavioral Data

Cognitive and behavioral symptoms have been shown in HD patients years before clinical diagnosis (35–37), and, more specifically impairments in WM and processing speed have been demonstrated.

Here, earlyHD patients performed significantly less accurate in the *n*-back task compared with both the HC and preHD group, indicating an early impairment of WM function. With regard to reaction time, both preHD and early HD were significantly slower compared with HC. Here, the disease might already have affected speed in the preHD group without concomitant accuracy loss. In the studies by Wolf et al. (9, 38) and the Image-HD study (8, 13), there were no significant behavioral effects between controls and preHD, an incongruence which may be explained by a larger sample size and thus more statistical power in our study. In Visit 1, Klöppel et al. (18) found longer (albeit not significant) response times in preHD than in controls in the same cohort.

### WM-Related fMRI Results

General linear model contrasts did not reveal group differences in the most prominent regions of the WM network that were revealed by the main effect of task, namely, bilateral DLPFC, bilateral IPC, and ACC, which were used to define the DCM. This is in contrast to earlier studies on WM with preHD and earlyHD participants, where complex patterns of increased and decreased brain activation were demonstrated (8, 9). However, there are some methodological differences that may have contributed to the diverging results. Here, we reported group differences FWE corrected for the whole brain, while Georgiou-Karistianis et al. (8) and Wolf et al. (9) reported results corrected for the more liberal cluster level. Furthermore, there were considerable differences in the respective WM paradigms. Georgiou-Karistianis et al. (8) used a different *n*-back task, and Wolf et al. (9) used the Sternberg item recognition paradigm (39). Finally, there were differences in the study sample in terms of clinical measures and sample size.

The absence of increased activation in mutation carriers in those regions corresponds to the findings from cross-sectional analyses of the Visit 1 data published elsewhere (18). Hence, differences in performance in mutation carriers compared with HC were not mirrored in differential increased or decreased brain activation. As between-group differences were assessed across the whole brain and were not based on ROI, emerging differences in activation might have been too subtle to be detected at a rigorous statistical threshold. With a more fine-grained analysis within the cortical WM network using DCM, we were able to show that the absence of differences in the GLM analysis was not representative for the processes taking place in mutation carriers’ brains during the WM task, but that in fact, there were numerous differences in connectivity between groups.

### WM-Related DCM Results

First, we were able to show that our fully connected DCM comprising five ROI derived from GLM results constituted a valid model of connectivity across all participants, which was further validated by testing the model between groups.

Moreover, 16 of 21 intrinsic connections within the model remained significant after testing against 0. Between-group analyses of DCM parameters revealed an interesting pattern of group differences (Figures 3A–D). preHD showed both increases and decreases in connectivity compared with HC. This result does not mirror the insignificant GLM findings concerning the cortical WM network and points to the potential of DCM to reveal more fine-grained differences.

The most striking similarity across all pairwise between-group comparisons of DCM parameters is the decrease in coupling towards right DLPFC in HD mutation carriers, which concerns both intrinsic as well as task-related connectivity. preHD showed a decrease in coupling toward right DLPFC in intrinsic connections only, while the comparison between earlyHD and HC revealed an additional decrease in task-related connectivity toward rDLPFC. Hence, when WM load increased from 1-back to 2-back, this resulted in diminished coupling in HD mutation carriers. Even when comparing premanifest and manifest carriers, there was a significant decrease in task-related coupling from ACC to rDLPFC in earlyHD. This result was complemented by diminished intrinsic coupling toward bilateral IPC in earlyHD, revealing an overall deterioration of connectivity in anterior as well as posterior parts of the WM network in manifest patients.

Our DCM results with regard to the rDLPFC complement previous findings obtained from time-course analyses of DLPFC activity in a smaller sample comprising HC, preHD, and earlyHD, who performed a different *n*-back task (12). They found a diminished signal in DLPFC in earlyHD at higher WM load (2-back), i.e., when task difficulty increased beyond capacity. This supports the interpretation of detectable deterioration in rDLPFC connectivity in premanifest and early manifest stages of the disease. Moreover, Gray and colleagues (40) found an association between reduced accuracy in a shifting response-set task and reduced prefrontal responsivity, underlining the association of DLPFC with diminishing performance.

In line with previous findings (41, 42), caudate volume was significantly compromised in mutation carriers and even more so in HD patients; however, there was no significant correlation between caudate volume and parameters of DCM connectivity. Furthermore, we were not able to identify significant correlations between 1-back or 2-back accuracy and connectivity, hence we cannot unambiguously connect weaker performance in mutation carriers with diminished connectivity. The intrinsic connection from ACC to right DLPFC was the only connection that was significantly correlated with a measure of task performance, namely, reaction times in the 2-back condition (Figure 3F). As this connection was more pronounced with slower response, one could argue that participants who struggled with the 2-back task already showed an alteration in intrinsic (not task-related) connectivity up front, an effect that was not revealed by our GLM analyses. The scatter plot in Figure 3F reveals that this correlation was not driven by slower response in the earlyHD group, but that response times show high variability across both premanifest and manifest mutation carriers. Hence, we conclude that connectivity toward right DLPFC, and more specifically cingulo-frontal effective connectivity, might be considered to be a sign of performance deterioration in the mutation carriers.

Taken together, we have shown that assessing effective connectivity with DCM may provide an added value to standard GLM analysis in the characterization of subtle differences at premanifest and early manifest stages of HD.

Considering the limitations of this study, the study sample comprised only a small number of earlyHD subjects compared with preHD and controls. While cluster analysis has previously been successfully applied in Alzheimer’s disease (43, 44) and HD (20) to stratify patients and predict clinical outcomes, in this study, exploratory cluster analysis of DCM parameters did not yield meaningful clusters. Hence, we were not able to reveal higher-level patterns at a network level and could not explore the association between subgroups of mutation carriers and measures of performance and disease burden.

## Conclusion

To the best of our knowledge, this is the first task-based WM-related DCM study in HD. The TrackOn-HD project recruited a large and balanced sample, and therefore our results can be regarded as robust. Previous DCM studies were often conducted in smaller samples, with subgroups including less than 20 individuals, yielding limited generalization of results (45).

We have demonstrated that valid DCMs can be established for WM-related ROIs in HD mutation carriers and that DCM helps to identify differences in connectivity that extend GLM findings. Thus, we would encourage the usage of such effective connectivity methods to further explore other neurodegenerative diseases such as Alzheimer’s and its precursor mild cognitive impairment. Our cross-sectional results could be expanded to explorations of longitudinal connectivity patterns in HD toward a better understanding of disease progression and its effect on network connections. DCM enables to explore directed connectivity and yields a more fine-grained picture of group differences compared with GLM and functional connectivity assessments. Therefore, we considered it to be a promising candidate to identify connections between brain areas of interest that could be targeted by tailored interventions.

## The TrackOn-HD Investigators

**A. Coleman, J. Decolongon, M. Fan, T. Koren**, University of British Columbia, Vancouver; **C. Jauffret, D. Justo, S. Lehericy, K. Nigaud, R. Valabrègue**, ICM and APHP, Pitié-Salpêtrière University Hospital, Paris; **A. Schoonderbeek, E. P. ‘t Hart**, Leiden University Medical Centre, Leiden; **H. Crawford, S. Gregory, D. Hensman Moss, E. Johnson, J. Read, G. Owen, M. Papoutsi, C. Berna, A. Razi, G. Rees, R. I. Scahill**, University College London, London; **D. Craufurd**, Manchester University, Manchester; **R. Reilmann, N. Weber**, George Huntington Institute, Munster; **J. Stout, I. Labuschagne**, Monash University, Melbourne; **M. Orth, G. B. Landwehrmeyer**, Ulm University, Ulm; **D. Langbehn, H. Johnson, J. Long, J. Mills**, University of Iowa, Iowa.

## Ethics Statement

The study was approved by the Ethics Committees of the Institute of Neurology, UCL (London), the University of British Columbia (Vancouver), Pierre and Marie Curie University (Paris), and the University of Leiden (Leiden). All participants gave a written informed consent according to the Declaration of Helsinki.

## Author Contributions

Conception and design of the study; article revision and final approval: JL, LM, ST, JS, SK, and ES. Analysis and interpretation of the data: JL, LM, SK, and ES. Article draft: JL, ES, and SK.

## Conflict of Interest Statement

The authors declare that the research was conducted in the absence of any commercial or financial relationships that could be construed as a potential conflict of interest.
