# Supplementary material for: Working Memory-Related Effective Connectivity in Huntington’s Disease Patients
Source: Front Neurol. 2018 Jun 4;9:370. doi: 10.3389/fneur.2018.00370 (PMC5994408; doi:10.3389/fneur.2018.00370)
Supplement: Supplementary file 1 [file data_sheet_1.docx]

| **Table S1:** Differences in effective connectivity between HC, HD mutation carriers and early-HD patients (result from ANCOVA, significance level p<0.0014 after Bonferroni correction). | | | | | | |
| --- | --- | --- | --- | --- | --- | --- |
| **Univariate Tests** | | | | | | |
| Dependent Variable | | Sum of Squares | df | Mean Square | F | Sig. |
| Intrinsic connections |  |  |  |  |  |  |
| lIPC to lIPC | Contrast | ,688 | 2 | ,344 | 6,288 | ,002 |
|  | Error | 8,746 | 160 | ,055 |  |  |
| lIPC to rIPC | Contrast | ,018 | 2 | ,009 | ,097 | ,908 |
|  | Error | 15,064 | 160 | ,094 |  |  |
| **lIPC to lACC** | Contrast | 1,329 | 2 | ,665 | 6,990 | ,00123 |
|  | Error | 15,215 | 160 | ,095 |  |  |
| **lIPC to lDLPFC** | Contrast | 1,463 | 2 | ,731 | 9,588 | ,000 |
|  | Error | 12,203 | 160 | ,076 |  |  |
| rIPC to lIPC | Contrast | ,526 | 2 | ,263 | 6,597 | ,002 |
|  | Error | 6,382 | 160 | ,040 |  |  |
| **rIPC to rIPC** | Contrast | ,940 | 2 | ,470 | 10.151 | ,000 |
|  | Error | 7,406 | 160 | ,046 |  |  |
| **rIPC to lACC** | Contrast | 2,122 | 2 | 1,061 | 15,165 | ,000 |
|  | Error | 11,196 | 160 | ,070 |  |  |
| **rIPC to rDLPFC** | Contrast | 3,747 | 2 | 1,874 | 28,064 | ,000 |
|  | Error | 10.682 | 160 | ,067 |  |  |
| lACC to lIPC | Contrast | ,650 | 2 | ,325 | 6,593 | ,002 |
|  | Error | 7,889 | 160 | ,049 |  |  |
| **lACC to rIPC** | Contrast | 3,220 | 2 | 1,610 | 32,187 | ,000 |
|  | Error | 8,003 | 160 | ,050 |  |  |
| **lACC to lACC** | Contrast | ,744 | 2 | ,372 | 11,808 | ,000 |
|  | Error | 5,039 | 160 | ,031 |  |  |
| **lACC to lDLPFC** | Contrast | ,495 | 2 | ,248 | 6,873 | ,00137 |
|  | Error | 5,766 | 160 | ,036 |  |  |
| **lACC to rDLPFC** | Contrast | 5,959 | 2 | 2,979 | 48,436 | ,000 |
|  | Error | 9,842 | 160 | ,062 |  |  |
| **lDLPFC to lIPC** | Contrast | 1,582 | 2 | ,791 | 21,630 | ,000 |
|  | Error | 5,851 | 160 | ,037 |  |  |
| **lDLPFC to lACC** | Contrast | 1,020 | 2 | ,510 | 20.300 | ,000 |
|  | Error | 4,021 | 160 | ,025 |  |  |
| lDLPFC to lDLPFC | Contrast | ,117 | 2 | ,058 | 3,365 | ,037 |
|  | Error | 2,781 | 160 | ,017 |  |  |
| **lDLPFC to rDLPFC** | Contrast | 1,097 | 2 | ,548 | 22,575 | ,000 |
|  | Error | 3,887 | 160 | ,024 |  |  |
| **rDLPFC to rIPC** | Contrast | ,904 | 2 | ,452 | 13,762 | ,000 |
|  | Error | 5,257 | 160 | ,033 |  |  |
| rDLPFC to lACC | Contrast | ,895 | 2 | ,448 | 5,000 | ,008 |
|  | Error | 14,324 | 160 | ,090 |  |  |
| rDLPFC to lDLPFC | Contrast | ,775 | 2 | ,387 | 2,839 | ,061 |
|  | Error | 21,828 | 160 | ,136 |  |  |
| **rDLPFC to rDLPFC** | Contrast | 7,269 | 2 | 3,634 | 11,302 | ,000 |
|  | Error | 51,451 | 160 | ,322 |  |  |
| Modulatory connections |  |  |  |  |  |  |
| lIPC to lACC (2B) | Contrast | 2,173 | 2 | 1,086 | 1,400 | ,250 |
|  | Error | 124,191 | 160 | ,776 |  |  |
| **lIPC to lDLPFC (2B)** | Contrast | 16,027 | 2 | 8,014 | 11,353 | ,000 |
|  | Error | 112,941 | 160 | ,706 |  |  |
| rIPC to lACC (2B) | Contrast | 1,820 | 2 | ,910 | 1,764 | ,175 |
|  | Error | 82,507 | 160 | ,516 |  |  |
| **rIPC to rDLPFC (2B)** | Contrast | 11,046 | 2 | 5,523 | 8,645 | ,000 |
|  | Error | 102,216 | 160 | ,639 |  |  |
| lACC to lIPC (2B) | Contrast | 2,173 | 2 | 1,087 | 1,960 | ,144 |
|  | Error | 88,681 | 160 | ,554 |  |  |
| lACC to rIPC (2B) | Contrast | ,311 | 2 | ,156 | ,232 | ,793 |
|  | Error | 107,199 | 160 | ,670 |  |  |
| lACC to lDLPFC (2B) | Contrast | ,357 | 2 | ,178 | ,297 | ,743 |
|  | Error | 95,954 | 160 | ,600 |  |  |
| **lACC to rDLPFC (2B)** | Contrast | 8,054 | 2 | 4,027 | 6,982 | ,00124 |
|  | Error | 92,282 | 160 | ,577 |  |  |
| lDLPFC to lIPC (2B) | Contrast | 4,962 | 2 | 2,481 | 4,549 | ,012 |
|  | Error | 87,266 | 160 | ,545 |  |  |
| lDLPFC to lACC (2B) | Contrast | 1,825 | 2 | ,913 | 1,611 | ,203 |
|  | Error | 90.649 | 160 | ,567 |  |  |
| lDLPFC to rDLPFC (2B) | Contrast | 1,316 | 2 | ,658 | 1,081 | ,342 |
|  | Error | 97,369 | 160 | ,609 |  |  |
| rDLPFC to rIPC (2B) | Contrast | 5,145 | 2 | 2,573 | 5,205 | ,006 |
|  | Error | 79,080 | 160 | ,494 |  |  |
| rDLPFC to lACC (2B) | Contrast | ,274 | 2 | ,137 | ,450 | ,638 |
|  | Error | 48,745 | 160 | ,305 |  |  |
| rDLPFC to lDLPFC (2B) | Contrast | ,625 | 2 | ,313 | 1,177 | ,311 |
|  | Error | 42,478 | 160 | ,265 |  |  |
